# Optimal feedback improves behavioral focus during self-regulated computer-based work

**DOI:** 10.1038/s41598-024-53388-3

**Published:** 2024-02-07

**Authors:** Maria Wirzberger, Anastasia Lado, Mike Prentice, Ivan Oreshnikov, Jean-Claude Passy, Adrian Stock, Falk Lieder

**Affiliations:** 1 University of Stuttgart, Stuttgart, Germany; 2 Max Planck Institute for Intelligent Systems, Tübingen, Germany

**Keywords:** Human behaviour, Computer science, Software

## Abstract

Distractions are omnipresent and can derail our attention, which is a precious and very limited resource. To achieve their goals in the face of distractions, people need to regulate their attention, thoughts, and behavior; this is known as *self-regulation*. How can self-regulation be supported or strengthened in ways that are relevant for everyday work and learning activities? To address this question, we introduce and evaluate a desktop application that helps people stay focused on their work and train self-regulation at the same time. Our application lets the user set a goal for what they want to do during a defined period of focused work at their computer, then gives negative feedback when they get distracted, and positive feedback when they reorient their attention towards their goal. After this so-called focus session, the user receives overall feedback on how well they focused on their goal relative to previous sessions. While existing approaches to attention training often use artificial tasks, our approach transforms real-life challenges into opportunities for building strong attention control skills. Our results indicate that optimal attentional feedback can generate large increases in behavioral focus, task motivation, and self-control—benefitting users to successfully achieve their long-term goals.

## Introduction

Imagine you are a journalist, facing the deadline to submit your first editorial to a high-profile international magazine. You are well aware that you should stay focused on writing to meet the requirements, but you have a hard time concentrating. All of a sudden, the funny online video that your friend had sent you earlier seems far more appealing. Although watching the video feels more worthwhile at this moment, you know that staying focused on writing would result in more beneficial outcomes for you in the long run. Eventually, your successfully published article might provide humanity with a unique piece of knowledge, boost your career trajectory, and increase your salary by a great deal. Situations like this vividly demonstrate how challenging it can be for people to engage in self-regulatory strategies, even when it is to their advantage to do so^1^. Previous work suggested that people perform much better when what is best for reaching their goals in the long run is also most appealing in the short run^2^. So, how can we make staying focused more enjoyable than getting distracted, help people internalize its benefits, and cultivate the motivation and capacity to follow through on their decision to get something done?

As the initial example indicates, the omnipresence of distracting information has become an inherent part of our everyday lives. Broader societal and economic implications range from students increasingly struggling to reach their educational goals, and employees suffering in mental well-being from spending their working hours ineffectively, not meeting their indicated progress goals, and then running up against impossible deadlines, to monetary loss resulting from constant interruptions at work. Distractions by social media cost the U.S. economy around $650 billion per year^3^. Building on theoretical models of interruption and resumption, these costs arise not only from the time spent on engaging with the distracting information but also from the time span needed to resume a task after getting distracted^4^. During this so-called resumption lag, information related to the interrupted task decays, which results in a more difficult access of prior goals and related task information^5,6^. To cope with these challenges, recent advances in user-centered technologies addressed the issue of working focused and productively on the computer, for instance by categorizing the user’s activities and/or blocking distracting content (see^7,8^ for recent systematic reviews).

Looking into the current landscape of digital self-control tools, for instance, the FlowLight system^9^ provides visual feedback on knowledge workers’ focused states. It operates via an external physical device that emits either red, yellow, or green light to signal individual states of interruptibility to co-workers. While the system gives implicit feedback on focus performance, thereby building awareness for one’s own productive states, existing evidence indicates that self-interruptions are more prone to impair task performance compared to external interruptions^10^. The physical Knob device^11^ adopts the reported light color scheme^9^ to signal users’ concentration states but adds a self-regulated blocking functionality to cover both external and self-interruptions. In a similar vein, this approach only gives indirect productivity-related feedback and further lacks a dedicated training on self-awareness. Preliminary evaluations with a very small sample of four participants hinted on positive effects regarding acceptance and reduction of disruptions.

Feedback on time allocation related to computer-based applications used is provided by the software meTime^12^, although the application does not include a classification of application use into focused or distracted times and no explicit goal setting beforehand. Evaluation results showed an improvement regarding users’ efficiency in time management, however, while they allocated less time to unproductive activities, they did not apply an efficient time allocation to productive activities. During semi-structured interviews, users expressed the demand for receiving a more explicit alerting feedback by the application. The widget TimeAware^13^ employes a self-monitoring visualization of time usage, emphasizing either productive or distracted times. Even though both conditions are set up in a semantically equivalent way, an improvement in productivity could only be observed for a distraction-emphasizing framing of the overall time spent. Even though results indicate an increased awareness of users’ productivity while using the tool, they did not hint on sustainable effects during the induced withdrawal phase. Again, this approach provides no direct feedback when users get distracted or distract themselves from their original task.

By contrast, further research evaluated a browser-based tool to enhance task focus in crowdwork by reminding users to focus when they resume from a self-interrupting unrelated task^14^. While the authors found such reminders to reduce task-switching frequency, these prompts do not provide feedback on resource investment related to reaching respective task goals. Building on these insights, the browser-based tool TimeToFocus^15^ provides feedback on the time spent interrupted on average during previously interrupted periods upon the start of an interruption. However, this immediate feedback does not refer to actual behavior but provides a summative reference to past behavior. Taken together, such tools can effectively assist across many situations^8^. However, neither of them trains underlying executive functions such as self-regulation and cognitive control skills in a systematic way, which, in the long run, could foster sustained performance, personal development, and cognitive growth.

Self-regulation generally refers to a broad selection of processes that guide individuals’ goal-directed behavioral, cognitive, metacognitive, and motivational activities^16,17^. Among these processes, cognitive control specifically relates to people’s ability to inhibit distracting impulses and redirect one’s attention to goal-relevant information^18^. Existing research already indicates that such skills can mitigate potentially harmful distraction effects in computer-based learning and working scenarios (e.g.^19^). This has proven to be essential in many aspects of day-to-day life, for instance, mental and physical health, school and/or job success and socioemotional development^20^.

Hence, strengthening these cognitive control skills by training can be considered a valuable investment (e.g.^21^). Systematic approaches to training cognitive skills are often designed as lab interventions with artificial tasks in artificial settings. While benefits of trained skills on strongly related domains, referred to as *near transfer*, are evident, clear evidence on far transfer, that is improvements in only weakly related domains, is still lacking^22,23^. Hence, if and how skills related to executive functions, such as self-control and attention control, can be improved in ways that benefit people in their daily lives remains a massive open question^24^.

Here, we introduce an alternative approach to improve people’s ability to resist distractions and temptations, allowing them to progress towards their chosen goals. Our desktop application supports people in staying focused on what they would like to do and trains their attention and self-control skills at the same time. This ensures that the training is relevant to the user’s daily life. Thereby, it transcends beyond blocking or removing programs and/or websites, which constitutes the most frequently employed set of strategies in existing digital self-control tools^8^. While employing both self-tracking and goal-advancement functionalities, such as recording performance history, displaying a timer, allowing for individual goal setting, comparing existing behavior with previously set goals, and reminding of these goals, we also introduce a dedicated reward mechanisms that values invested effort by indicating gain and loss of points—a feature only rarely present in existing solutions. Substantially extending prior work, our feedback touches upon higher mechanisms of self-regulation, namely, the value of invested effort toward reaching a self-defined goal. To get aware of and define one's own goals, we explicitly encourage a more comprehensive goal-setting process in the activity preparation phase.

Subsequently, we will describe core functionalities of our application and outline a multi-day field experiment, which we conducted to evaluate effects of our application on human performance in computer-based settings. Our findings indicate that participants were more focused on their tasks in terms of their actual behavior (*behavioral focus*), and they reported greater task motivation and increased abilities to cope with tempting distractions.

We take these findings as promising hints that our approach can eventually help people become more focused and productive. In the bigger picture, this might support them in reaching their long-term goals, such as career success or a healthy work-life balance.

## Materials and methods

### A desktop application that fosters self-regulation

The importance of sufficient feedback to foster skill acquisition has been widely demonstrated in decades of educational research^25,26^. Leveraging these insights, we developed a desktop application that can support people’s focused attention while working in computer-based environments.

#### Core functionality: goal setting and feedback

Following evidence from goal setting research (e.g.^27^), people are more likely to follow their goals when they are highly committed. Even though legitimate authorities or peers might exert a persuasive influence, commitment towards a goal is a personal choice, which increases with goal importance and the experience of being rewarded for it. Giving users agency to choose their own goal aligns with evidence from Self-Determination Theory (SDT^28^), which emphasizes that learners’ perception of competence, relatedness or autonomy can increase their task-related motivation. The enhancing effect of autonomy on performance has also been demonstrated in instructional design research. Prior work found not only less perceived effort in task execution but also increased learning performance when including a choice option in the learning task^29^. Decades of research further emphasize the moderating effect of feedback on goal-related performance, resulting in improved performance when combining both feedback and goal setting^27^. Our application therefore guides the user to set a goal for what they want to do during a defined period of work, subsequently called *focus session*, and then gives them feedback on how well they are pursuing that goal. Concretely, our application gives the user two types of feedback. During a focus session, the user receives feedback on whether they are currently distracted (*formative feedback*). After the focus session, the user receives feedback on their overall level of focus during the focus session (*summative feedback*).

**Figure 1:**
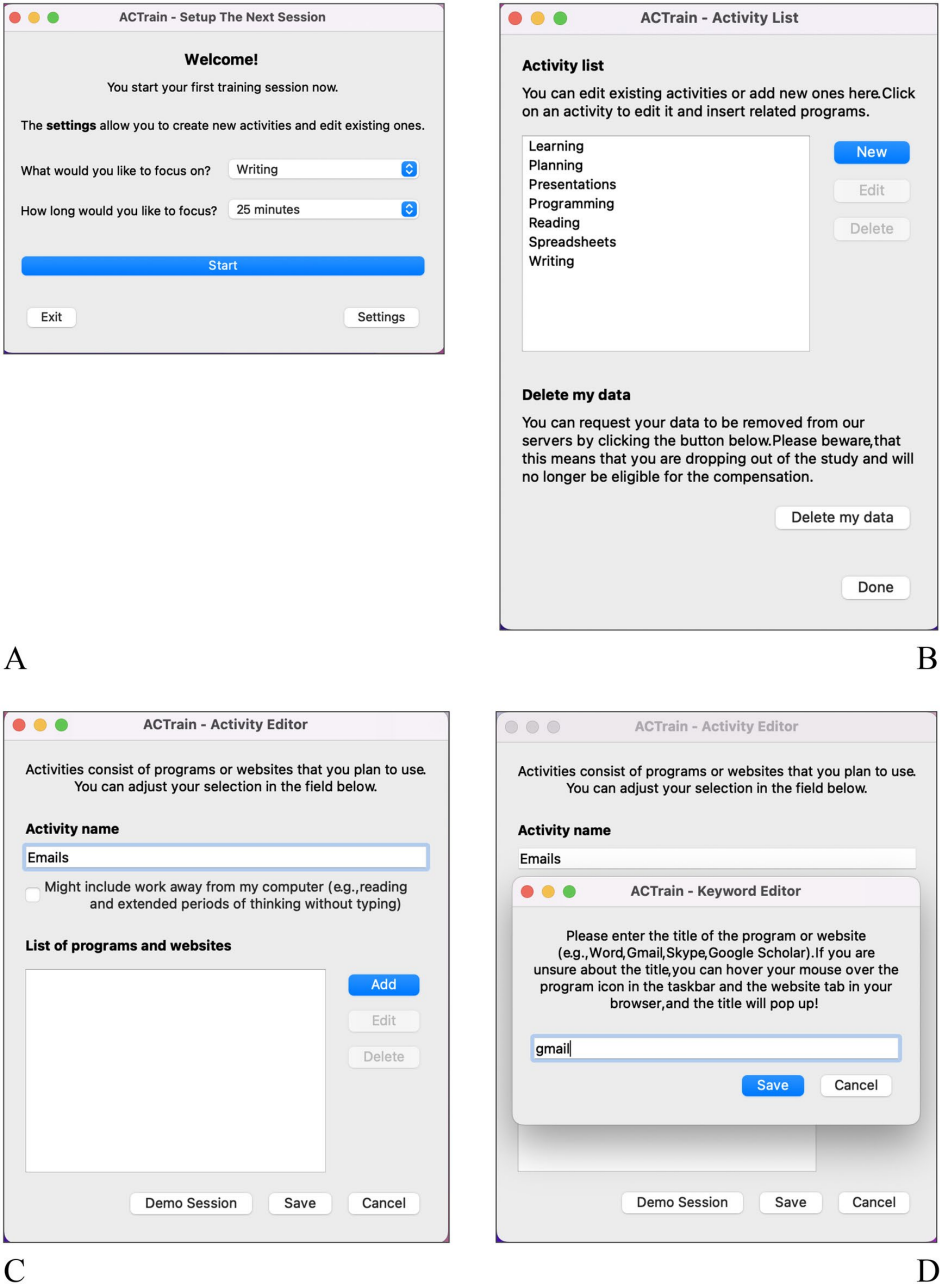
Basic configuration of sessions and related activities, showing start screen (**A**), list of potential activities (**B**), editor for new activities (**C**), and editor for activity-related keywords (**D**).

As Fig. 1A displays, a focus session starts with setting a goal for this session. In addition, the user can also select an individual focus session duration, aligning the software use with their own workflow. During a focus session, the software uses a keyword list derived from the selected activity to determine whether a user is in a focused or distracted state, that is, whether the programs and websites the user interacts with match their goal for the current focus session. Inactivity of mouse and/or keyboard for more than 2 min is also classified as distraction. However, for tasks such as reading or lecture video watching, including longer periods without mouse or keyboard activity can be defined as well by selecting the respective option in the settings menu (see Fig. 1C). Upon the first use of the software and/or the first time performing a new activity, this activity is planned down to the programs or websites required to complete the chosen task goal (see Fig. 1B–D). Thereby, the employed functionality accounts for the fact that complex tasks require more than one program to solve it, resulting in activities with a broader spectrum of related programs and websites.

**Figure 2:**
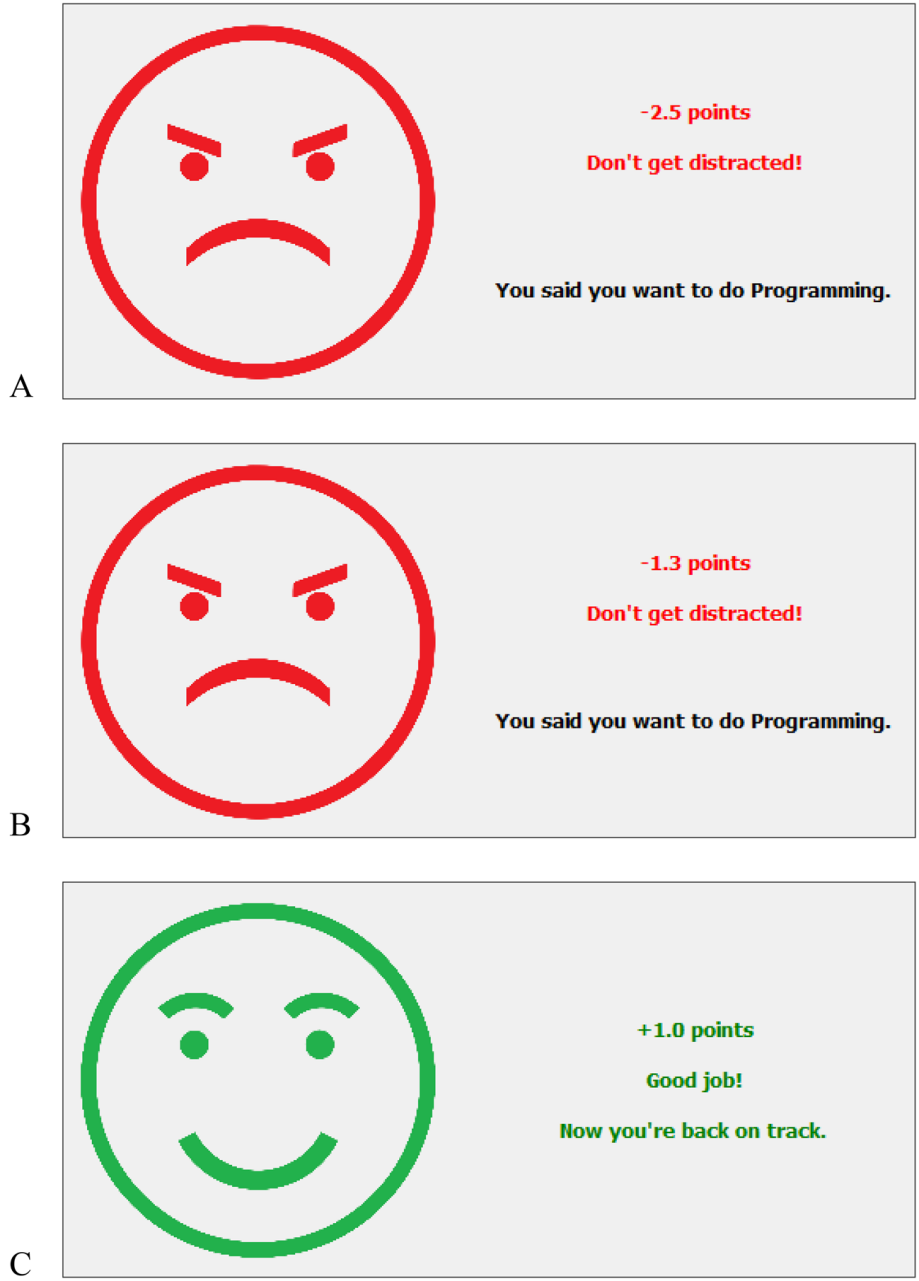
Formative feedback during focus sessions for getting distracted (**A**), staying distracted (**B**), and regaining focus (**C**).

Returning to our initial example of the journalist: If one—despite all good intentions—finds themselves watching an online video, our application would detect a deviation from your initially specified goal of writing, as you no longer use your indicated text editor, and display the negative feedback message shown in Fig. 2A. As you see, it reminds you of your own prior intention, holding you accountable for your individual achievements. As long as you stay distracted, our application will give negative feedback every 7 s (see Fig. 2B). Now, if the software successfully manages to convince you to return your focus to your initially chosen task, the detected congruence between the specified focus and the actually performed task results in positive feedback, displayed in Fig. 2C. According to^30^, feedback generally answers three questions: where I am going (*feed up*), how I am doing (*feed back*), and where to go next (*feed forward*). Our application’s formative feedback therefore reminds users of their original goal (*feed up*), indicating if the chosen focus is maintained or derailed (*feed back*), and hinting on the next action, i.e., getting back on track or staying on track (*feed forward*). It references the respective *task* by reminding the user of their initially set activity, provides information on the current state of *process*, i.e., staying focused vs. getting distracted, and fosters engaging in *self-regulation* activities in case of getting distracted.

Our rationale for designing this formative feedback was that choosing actions with higher long-term gains is much easier for people when those actions are also beneficial in the short run^2^. Given that our cognitive resources are limited, prior research shows that we intend to minimize cognitive costs as much as possible and align resource investment towards rewarding outcomes^31^. In the context of cognitive control operations, this ties in with the Expected Value of Control (EVC) theory^32^, which takes into consideration that the value of exerting cognitive control at one time also depends on future decisions. Considering demands in our daily life, we continuously need to decide whether or not we exert cognitive control to pursue a specific goal or let loose and get distracted, resulting in a series of correlated decisions over an extended period of time. In mathematical terms, following prior work^33^, we can formalize this sequence of decisions towards a selected goal as a finite-horizon Markov Decision Process (MDP^34^). A finite-horizon MDP is defined by a set of possible states that an agent can be in a set of actions the agent can take, conditional probabilities of transitioning from one state to another depending on the action taken, a reward function, and for how long a task is performed.

While similar research takes interruptions and future resumption costs into account^31^, we focus on costs of exerting cognitive control to resist tempting interruptions at all. Driven by shared similarities in underlying cognitive operations, we used a recently developed optimal feedback method^35^ to compute feedback signals that align how the user experiences (not) investing the mental effort required to pursue their goal with the value of doing so. Thereby, “optimal” refers to a metacognitively informed choice related to anticipated cognitive resource investment. Even though decision mechanisms investigated in this previous work were applied in a much simpler task environment, we consider them transferable to our context of self-regulated goal focus. Additionally, the promising results obtained^35^ raise the question of potential transferability and explanatory value related to more complex naturalistic task settings. Consequently, we used the Learned Value of Control (LVOC) model^33^ to simulate how well people would learn from such optimal feedback and plugged the resulting reward values into our software (for more details on related equations see supplementary material, section A1).

This feedback rewards each mental action (e.g., to return one’s focus to a goal-consistent activity) according to how much it helps or hinders the user in achieving their stated goal for the current focus session (e.g., to spend as much time on writing as possible). The resulting feedback messages therefore signal how many points the user lost or gained when they stayed focused (not displayed), got distracted (− 2.5 points; see Fig. 2A), remained distracted (− 1.3 points every 7 s; see Fig. 2B), and regained their focus (+ 1.0 points; see Fig. 2C), respectively. To increase the effect of the feedback, we included visual cues related to color and facial expression and added auditory cues, resembling an alarm for negative feedback, and gaining points in a video game for positive feedback. Feedback messages were always displayed for 5 s to allow the user to process the content but at the same time minimize distractibility of the feedback itself. By contrast, the feedback for staying focused was not shown immediately to avoid distracting the user constantly. However, it did inform the summative feedback that the user received at the end of a focus session.

**Figure 3:**
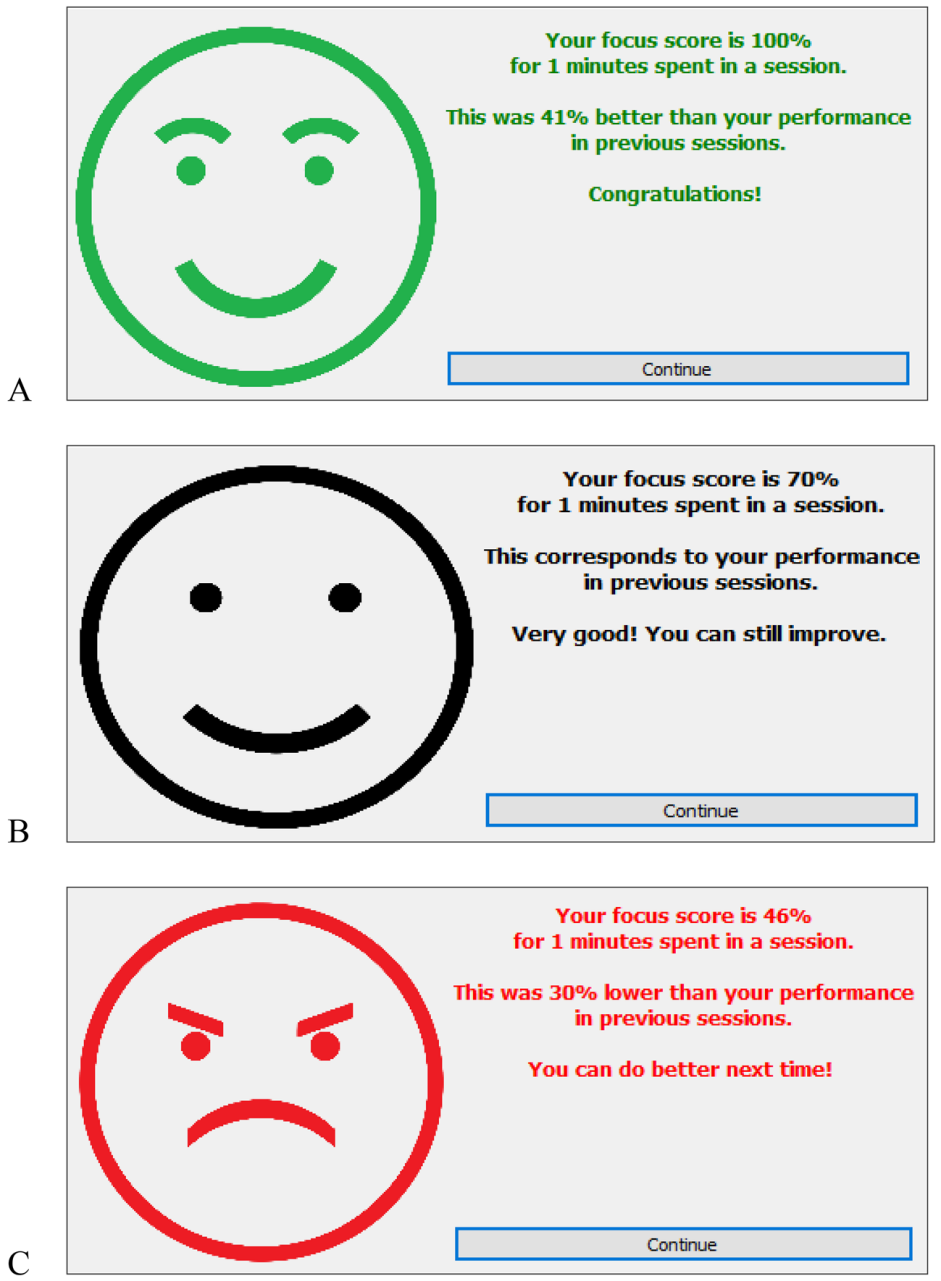
Summative feedback after completing a focus session, indicating a decrease (**A**), maintaining (**B**), or improvement (**C**) of focus performance in this session.

The summative feedback, displayed in Fig. 3, is based on the points the user earned in the focus session. Our application sums up the points the user gained or lost to compute a score that measures for how long the user stayed focused and how often and for how long they got distracted. This so-called *focus score* is stored after each focus session. Our implementation thereby used an earlier version of Equation 6 (see supplementary material, section A1), which builds on the maximally obtainable point score for a fully focused session and subtracts the point values of the user’s state transitions from focused to distracted, distracted to focused, and from distracted to distracted. We further assume that systematic changes in the focus score across sessions reflect changes in the person’s underlying ability to stay focused on the chosen task. However, the focus score can also fluctuate at random. Our software therefore uses a Kalman filter^36^ to smooth out these random fluctuations and obtain a reliable estimate of practice-induced improvements and lapses in the user’s ability to stay focused. When the estimated change in the user’s ability to stay focused is positive, then the user receives positive summative feedback (see Fig. 3A); when their inferred ability remained the same, then the summative feedback is neutral (see Fig. 3B); and when the inferred change in the user’s ability to stay focused is negative then the summative feedback is negative (see Fig. 3C). For further details, see section A2 in the supplementary material.

#### Usability ratings

Software development followed an iterative user-centered design process with embedded user studies that focused on technical functionality, usability, and user satisfaction. A total of 35 volunteers (*M*_age_ = 27.75 years, *SD*_age_ = 5.37, 43% female) participated in usability studies between April and December 2020. As part of the evaluation, they received defined use case scenarios, such as writing a text or creating a presentation. In addition, they could perform self-selected tasks with the software. We used think-aloud protocols and screen recording and administered the meCUE 2.0 usability questionnaire^37,38^.

The meCUE 2.0 comprises five modules that break down into ten subscales to assess defined qualities of experience related to using a technical product. The first module relates to perceived usefulness (e.g., “With the help of this product I will achieve my goals.”) and usability (e.g., “It is quickly apparent how to use the product.”), whereas visual aesthetics (e.g., “The product is creatively designed.”), status (e.g., “The product would enhance my standing among peers”), and commitment (e.g., “I could not live without this product.”) form inherent dimensions of the second module. Positive emotions (e.g., “The product makes me feel happy.“) and negative emotions (e.g., “The product annoys me.“) related to the product are addressed in the third module, and the fourth module involves the aspects of product loyalty (e.g., “I would not swap this product for any other.“) and the intention to use the product (e.g., “If I could, I would use the product daily.“). Module five involves a rating scale asking for an overall impression of the product (“How do you experience the product as a whole? “). The average scores across modules and sub-dimensions are displayed in Table 1.

**Table 1:** User evaluations with the meCUE 2.0 combined over studies from April to December 2020.

Module	Subscale	M	SD
Module I	Usefulness^a^	4.55	1.37
	Usability^a^	5.44	1.04
Module II	Visual Aesthetics^a^	2.80	1.15
	Status^a^	3.35	1.46
	Commitment^a^	2.14	1.10
Module III	Positive emotions^a^	3.27	1.45
	Negative emotions^a, c^	3.06	1.28
Module IV	Intention to use^a^	3.20	1.50
	Product loyalty^a^	3.41	1.39
Module V	Overall evaluation^b^	15.33	4.40

^a^Likert scale ranging from 1 to 7 in line with the meCUE 2.0.

^b^Scale ranging from 0 to 20 due to technical implementation, deviating from scale range of − 5 to 5 in the meCUE 2.0.

^c^Higher values are related to more negative emotions.

Upon evaluating these scores with reference to the respective scale means, we find that overall, our application receives a quite reasonable evaluation score. Inspecting the subscales in more detail, we observe that our application is perceived as both useful and usable, obvious from above-average scores in both subscales. In addition, looking at both subscales in Module III, we find scores below average, indicating that our application does not elicit strong emotional responses that might interfere with performance. Improvements persist, for instance, regarding visual design, taken from the rather low score of visual aesthetics and the around average score of perceived status. We also conclude quite limited implications for commitment, intentions of sustained use and product loyalty due to the short and one-time setting of the user studies. Taken together, these findings suggest that our application could be useful for tackling the real-life challenge of staying focused on a chosen task.

### Field experiment

Building on the encouraging insights obtained from the reported small-scale iterative formative evaluations, we proceeded to evaluate our application in a large-scale field setting. Addressing the need for control group comparisons in the field of digital self-control tools^8^, we conducted a randomized controlled field experiment with a longitudinal design to investigate whether our application can improve people’s task-specific performance, feedback-induced learning, and general executive functions.

#### Hypotheses

First, we predicted that training-related performance improvements with optimal feedback would result in people spending a higher proportion of their time focused on productive activities and a lower proportion of their time focused on distracting activities. This should also improve people’s self-evaluations of their productivity, progress towards their goals, and motivation to perform their task. With increased self-control, people might also experience weaker and less challenging temptations for getting distracted and resist distractions more successfully. Second, we hypothesized that improved learning would manifest in faster improvements in participants’ ability to stay focused on their chosen goal, as well as in their self-reported productivity, self-control, and task motivation. In addition, we expect temptations to do other things during the focus session to become less frequent, weaker, and less challenging. Moreover, people's ability to resist the temptation to do other things during their focus session should improve with training. Third, we hypothesized that training-induced improvements in people’s general executive functions would lead to higher scores on a standardized test of cognitive control, better self-control, and higher perceived self-efficacy. In summary, we had three main hypotheses:

##### H1

Receiving optimal feedback increases people’s performance on their self-chosen tasks.

##### H2

Optimal feedback enhances people’s learning of attention control skills.

##### H3

Training attention control with optimal feedback improves people’s general executive functions more than practicing without feedback.

#### Experimental design

Using a set of pre-posttest and process-related measures, our experiment compared the full software with optimal feedback (experimental condition) to a placebo version without feedback (control group). The software version used by the control condition also offered the opportunity to conduct training sessions and specify activities with related programs. However, the control group received no feedback; it was only shown neutral information on the length of the completed session in the end. The application used in this experiment can be downloaded at https://osf.io/8f6hx/.

The entire study and plan for data collection and analysis was approved by the ethics committee of the Medical Faculty at the University of Tübingen, Germany (project number 119/2019BO2). All participants provided informed consent and the research was performed in accordance with the relevant guidelines and regulations outlined in Standard 8 of the Ethical Principles and Code of Conduct for Psychologists^39^.

#### Participants

We recruited a sample of 235 volunteers from Prolific, a university mailing list, and relevant social media groups (*M*_age_ = 24.69, *SD*_age_ = 5.08, 54.9% female). Necessary exclusion criteria during recruitment were: (a) red-green blindness; (b) participation in previous studies with the software. All involved participants already had a university degree or were enrolled at the university to obtain a bachelor’s degree. 67.7% of participants reported a daily computer use of more than 5 h. The majority of our participants (81.1%) preferred Windows and 18.9% preferred MacOS. After completing the study procedure, participants received a 35€ gift card or 35€ payment as a compensation.

**Figure 4:**
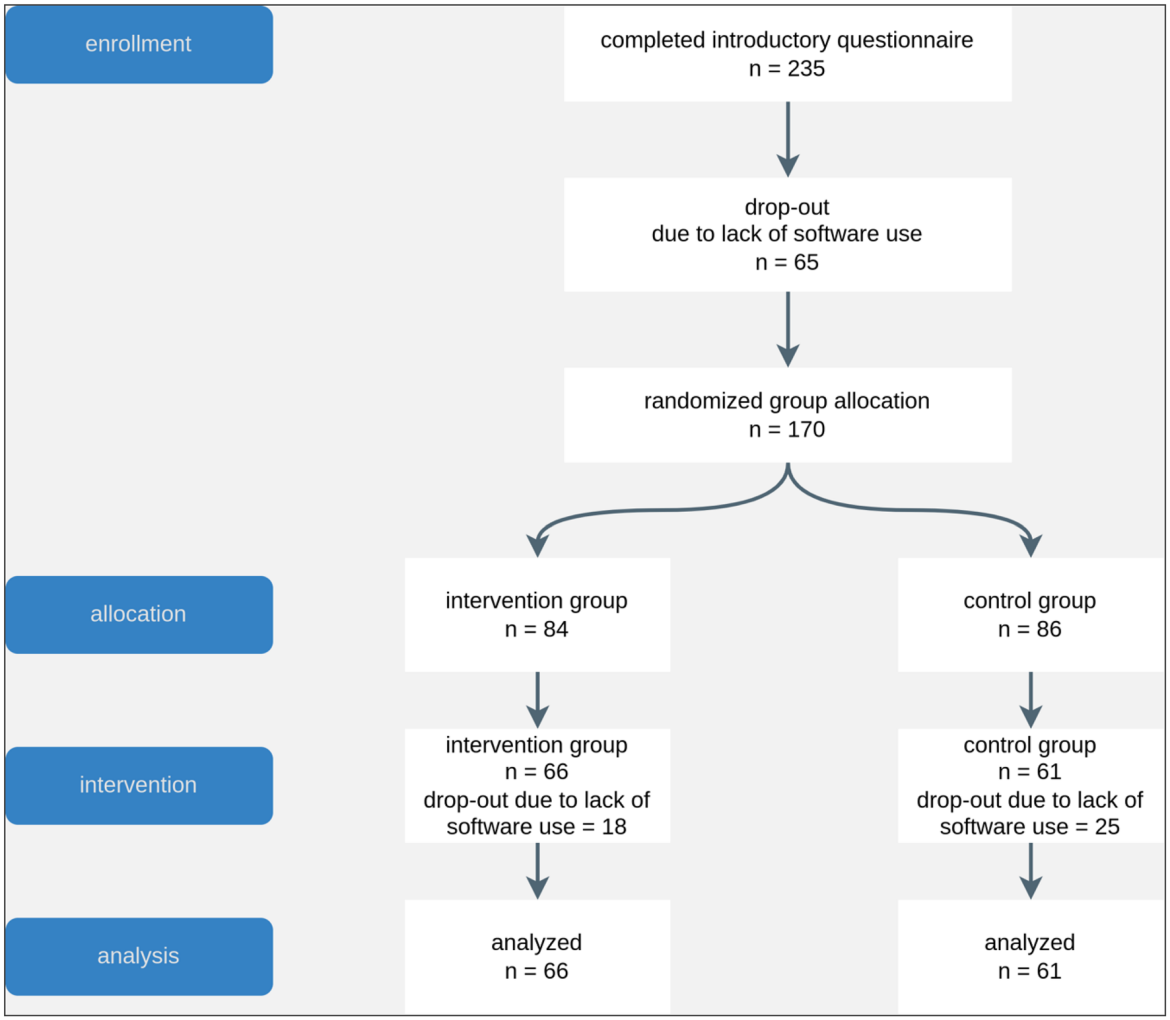
Flow diagram of study enrollment and subsequent participant dropout across group allocation, intervention, and analysis stages.

Due to dropout at different stages of the study (see Fig. 4), only 127 participants provided data across all stages of the study, i.e., pre-survey data, session-level software interaction data as well as the post-survey (*M*_age_ = 24.57 years, *SD*_age_ = 5.13, range 18–46, 56.7% female). On average, these participants completed 15.47 computer-based training sessions (*SD* = 13.42), with an average duration of 32.25 min per session (*SD* = 19.94.). Almost two thirds of the participants (58%) trained 10 h or more (*M* = 9.94, *SD* = 6.55, range 0.08–40.53).

As obvious from Fig. 4, our observed dropout of 108 participants (45% out of everyone completing the introductory questionnaire) by the post-survey is similar to dropout rates reported for other internet-based longitudinal field experiments^40^. Upon deeper investigation, most of the dropout (~ 60% out of everyone dropping out across various stages) occurred between the pre-survey and the start of the intervention, which did not involve using our application. Therefore, only 170 participants started the study. Taking a closer look at this proportion of participants only, the dropout rates did not differ systematically between the experimental condition (21%) and control condition (29%), χ^2^(1) = 0.940, *p* = 0.332.

#### Measures

The inspected set of outcome measures involved two sets of outcome variables: pre-post measures (sustained attention, self-control, goal progress) and outcomes that were measured throughout the intervention (session metrics, experience sampling). These break up into three groups of measures related to task-specific performance, feedback-induced learning, and general executive functions.

*Session metrics.* For each focus session, our application recorded the chosen activity (including assigned programs and websites), intended and actual session durations, the focus score (calculated from the points gained for time spent focused and regaining focus, and the points lost for getting distracted and time spent distracted; see Equation 6 in the supplementary material, section A1), and people’s self-rated productivity (queried by “How do you rate your productivity in the completed session?”) on a seven-point Likert scale (ranging from “very, very low” to “very, very high”).

*Experience sampling.* Our software further included an experience sampling module, which we used to measure participants' self-control skills. Inspired by prior work^41^, the dialog asked the user to report the number of temptations to do something other than the intended tasks. Our application further asked the user to indicate how strong and challenging these temptations had been perceived, their ability to resist these temptations and their general motivation to perform the chosen task. Except for the number of temptations, for which users were asked to enter a numeric value, they indicated their responses on seven-point Likert scales. About 75% of all focus sessions were accompanied by an experience sampling dialogue, which was displayed either before or after a session. The included questions referred to either the just completed focus session or the last hour of work before starting that focus session.

*Sustained attention.* To measure general capacities for sustained attention, we used a continuous performance task (CPT)^42^. In the AX-CPT variant^43^, participants respond by pressing one button when a target is presented, and another button when any other stimulus is presented. Usually, the target letter is X. However, there is an added constraint: X is only a target when it is preceded by the letter A. Thus, if a participant sees A–X–A–X, both X's are targets. If a participant sees B-X-B-X, neither of those X's are targets. Researchers can not only manipulate how likely it is that a target appears, but they can also manipulate the context in which stimuli appear. In particular, when A–X pairs are common, the participant will have a prepotent tendency to respond to the stimulus that follows the A, expecting it to be a target. But sometimes it is a Y or some other letter, and the participant must then inhibit that prepotent tendency to respond. Therefore, the AX-CPT measures both a person's ability to remember a goal (i.e., to detect A–X pairs), and their ability to process context (e.g., knowing that if a B is presented, the next letter cannot be a target, or if an A is presented, the next letter is likely a target). Relating to the initially outlined characteristics present in knowledge work, we regard the validated standardized task framework of a CPT to be an indication of prerequisites for far transfer regarding underlying inhibition and response mechanisms.

*Self-control.* In addition, we used the Brief Self-Control Scale^44^ in both the pre- and post-survey to assess participants' self-control skills. This standardized scale targets the construct at trait level with 13 items on a five-point Likert scale (“not at all like me” to “very much like me”). The included items relate to five underlying factors: general capacity for self-discipline (e.g., “I am good at resisting temptations.”), inclination toward deliberate or non-impulsive actions (e.g., “I often act without thinking through all the alternatives.”), healthy habits (e.g., “I have a hard time breaking bad habits.”), self-regulation in service to build a strong work ethic (e.g., “Pleasure and fun sometimes keep me from getting work done.”), and reliability (e.g., “I am able to work effectively toward long-term goals.”).

#### Procedure

In both conditions, the experiment span twelve days. On the first day, all participants were asked to complete a pretest survey, the AX-CPT task, and set a goal for themselves they wanted to accomplish during the study. From the fourth to ninth day, participants were encouraged to complete a total of 10 h of training with our application. On the final twelfth day, people completed a posttest survey—including the option to upload logfiles from the attention training software—and the AX-CPT task. In between, from the first to third and tenth to twelfth day, participants only used a commercial time tracking software, which is not further considered here (see supplementary material, section A3).

## Results

On average, participants completed 18.59 focus sessions in the experimental group (*SD* = 14.18, range 1–78), which is a slightly higher number compared to the control group (*M* = 15.80, *SD* = 10.05, range 1–53). By contrast, participants in the experimental group displayed slightly shorter focus sessions on average (*M* = 32.01, *SD* = 26.74, range 1–60), compared to the control group (*M* = 36.74, *SD* = 31.99, range 1–60). Taking a deeper look into activities reported, we saw that 75% of all activities were work or study-related, with 23% of activities related to writing, 17% related to reading and the remaining 35% being difficult to categorize. With 14% of reported activities, language learning has been a prominent category as well, while 4% of reported cases relate to entertainment-related activities, and the remaining 7% fall into the category of miscellaneous.

Approaching our hypotheses at a statistical level, we divided them into three families: task-specific performance (eight tests), focus-induced learning (seven tests), and improvements in overall executive function (four tests). Our hypotheses and analysis plans were pre-registered (https://osf.io/49nqd and https://osf.io/hxytu). Deviations from our pre-registered analysis plan are reported in the supplementary material in section A3. We controlled the false discovery rate of all tests within a given family according to the Benjamini–Hochberg procedure^45^ as implemented in *p. adjust* in the R stats package^46^. The following three sections present the results for each of these three families of hypotheses in turn.

### Task-specific performance

The first set of analyses comprised eight tests comparing the experimental group against the control group on different measures of how well they performed the tasks they had set out to do in their focus session (task-specific performance). Seven of the tests were independent samples t-tests comparing the experimental and control conditions on the focus score computed by the attention training software, self-reported scores for productivity and task motivation, and self-reports of resisting temptations, challenges in resisting temptations, perceived temptation strength, and number of experienced temptations. The eighth test was a residualized change regression (see^47^) in which self-reported goal progress at the end of the study was predicted by goal progress at pre-test and a dummy-coded condition variable. Some example goals were to “Finish the paper I want to submit,” “To be a better version of myself,” and “I want to learn as much new information as I can.” All tests reported here and in subsequent sections are two-tailed, in part to leave open the possibility of discovering backfire effects. Of these eight tests, six were significant after adjusting for false discovery rate. All significant differences pointed in the predicted direction, as reported in Table 2. In brief, participants who were supported by our application outperformed the control group on all measures of task-specific performance except for the number of temptations they experienced during their working sessions and their overall progress towards the longer-term goal they had set at the beginning of the study.

**Table 2:** Summary of hypothesis tests for task-specific behavior (hypothesis family one).

Variable	Mean experimental	Mean control	Estimated difference d	t	*p*	df	95% LCI of d	95% HCI of d	Cohen’s d
Focus score	299.95	126.35	173.61	6.73	< 0.001	110.73	122.48	224.73	1.20
Productivity	5.63	5.09	0.54	3.41	0.003	112.77	0.86	0.23	0.61
Task motivation	5.46	4.99	0.47	2.78	0.009	119.63	0.81	0.14	0.50
Temptation resistance	5.27	4.62	0.65	3.35	0.003	119.77	1.04	0.27	0.60
Temptation challenge	3.14	3.69	− 0.55	− 2.82	0.009	119.46	− 0.16	− 0.94	− 0.51
Temptation strength	3.28	3.78	− 0.50	− 2.61	0.012	119.78	− 0.12	− 0.88	− 0.47
Temptation number	1.70	2.16	− 0.45	− 1.65	0.101	116.11	0.09	− 1.00	− 0.30
Goal progress	13.65	16.02	− 2.36	− 1.53	0.130	124.00	− 2.33	0.30	− 0.25

The first seven tests are Welch’s independent samples t-tests. The eighth test, on goal progress, is a residual change regression of experimental condition on post-study goal progress, controlling for pre-study progress. Cohen’s *d* values were computed with the adjusted *df*s from the Welch t-tests.

**Figure 5:**
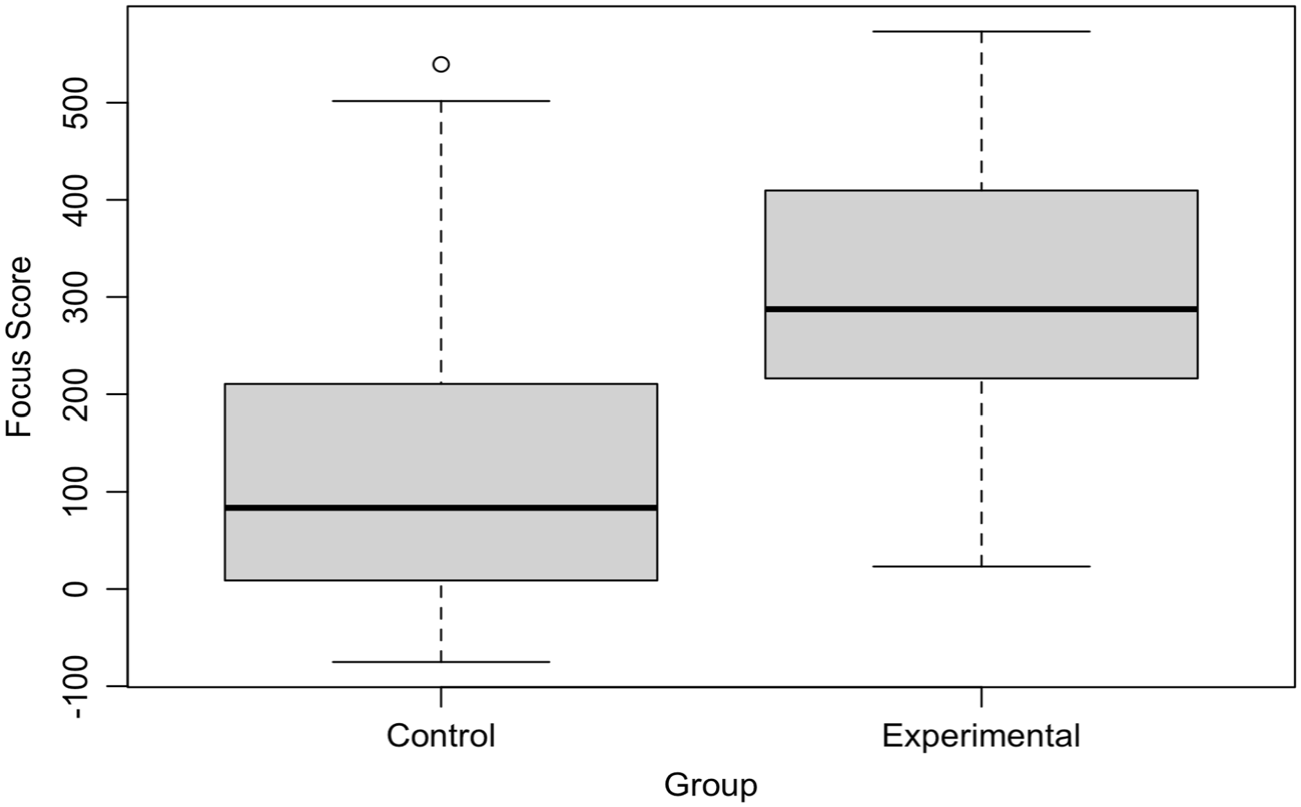
Boxplot of mean focus scores in both control and experimental conditions.

Crucially, participants in the experimental condition exerted significantly more focused behavior related to their tasks than those in the control condition, see Fig. 5. With Cohen’s *d* = 1.20, this effect was very large^48^. Furthermore, participants in the experimental condition rated themselves as significantly more productive and task-motivated and reported significantly lower challenge, strength, and number of temptations and a greater ability to resist temptations than those in the control condition. These effects tended to be in the moderate to large range with *d* absolute values from 0.25 to 0.61. In summary, our software produced greater actual focus and increased participants’ self-reported productivity, task motivation, and abilities in handling distractions in the experimental condition compared to the control condition.

### Feedback-induced learning

Our second family of analyses, including seven tests, related to feedback-induced learning in terms of focus performance, self-reported productivity, and task motivation as well as the self-reported number, strength, and challenge of temptations and participants’ resistance towards them. These tests were conditional growth curve models, in which we examined differential growth by condition for the same set of variables submitted to the t-tests above. We used the Nelder-Mead optimization of parameters^49^, restricted maximum likelihood (REML) estimation of variance components^50^, and Satterthwaite’s approximation for computing degrees of freedom^51^.

For these tests, the crucial effect was the interaction between condition and time—represented by either the session number or the number of the displayed experience sampling dialog—as this would indicate differential growth over time between groups. After adjusting for false discovery rate, we did not observe significant interaction terms for the focus score (β = 2.30, 95% CI [− 0.26, 4.86], *SE* = 1.27, *t*(44.09) = 1.81, *p* = 0.269), self-rated productivity (β = 0.00, 95% CI [− 0.02, 0.02], *SE* = 0.01, *t*(28.05) = 0.26, *p* = 0.855), or task motivation (β = 0.03, 95% CI [0.00, 0.07], *SE* = 0.02, *t*(97.65) = 2.08, *p* = 0.269).

Conditional growth curve models for temptations’ number, strength, challenge and resistance further controlled for participants’ self-control and accuracy achieved in the AX-CPT. Likewise, the condition over time interaction for the number (β = − 0.00, 95% CI [− 0.05, 0.04], *SE* = 0.02, *t*(86.50) = − 0.18, *p* = 0.855), strength (β = − 0.03, 95% CI [− 0.07, 0.01], *SE* = 0.02, *t*(60.01) = − 1.29, *p* = 0.471), and challenge of temptations (β = − 0.01, 95% CI [− 0.06, 0.03], *SE* = 0.02, *t*(71.25) = − 0.49, *p* = 0.855) as well as participants’ resistance towards temptations (β = 0.02, 95% CI [− 0.02, 0.05], *SE* = 0.02, *t*(91.24) = 0.89, *p* = 0.661) lacked significance. This pattern is indicative of the absence of a learning effect from receiving feedback when interacting with our attention training software.

To discern whether the behavioral improvement of participants happened solely due to the presence of the feedback, we performed an exploratory investigation if the number and length of distractions, captured by the number of times the application logged focus reminder events when participants stayed distracted, changed throughout the study. The investigation encompassed splitting individual focus sessions into the three time bins related to early, middle, and late stages of participation. As that the number of completed sessions highly differed between participants, we allocated 33% of all focus sessions to each category. We assumed that in the later focus sessions during our intervention, participants would exhibit fewer distractions and reduced distraction durations, thereby elucidating the specific influence of feedback on behavioral outcomes. Looking at the descriptive statistics, we can generally observe a substantially lower number of focus reminder events in the experimental group (*M*_*early*_ = 7.13, *SD*_*early*_ = 35.70; *M*_*middle*_ = 2.38, *SD*_*middle*_ = 10.50; *M*_*late*_ = 2.68, *SD*_*late*_ = 15.60) compared to the control group (*M*_*early*_ = 119.00, *SD*_*early*_ = 154.00; *M*_*middle*_ = 174.00, *SD*_*middle*_ = 186.00; *M*_*late*_ = 162.00, *SD*_*late*_ = 184.00) across all stages. These results further hint on performance improvements in the middle and late stages in the experimental group, whereas we observe a reversed trend in the control group.

Consequently, to test for statistical significance of these observed differences, we used a linear mixed-effects model including the number of focus reminder events as dependent variable and the dummy coded session order, condition, and interaction between both variables as well as a random intercept for participants as independent variables. We applied the same approaches to parameter optimization, estimation of variance components, and computing degrees of freedom as outlined before. Both the interaction for experimental group and middle sessions (β = − 56.80, 95% CI [− 74.62, − 38.99], *SE* = 9.08, *t*(2062.67) = − 6.25, *p* < 0.001) as well as the interaction between experimental group and late sessions (β = − 39.61, 95% CI [− 56.94, − 22.27], *SE* = 8.84, *t*(2065.13) = − 4.48, *p* < 0.001) were statistically significant. This confirms that, compared to early sessions, being in a middle or late session indeed corresponds to fewer and shorter distractions in the experimental group compared to the control group.

### Improvements in overall executive functions

Finally, hypothesis set three concerned improvements in overall executive functions in terms of pre-post changes in behavioral accuracy and reaction time in the AX-CPT as well as self-reported self-control and self-efficacy. These hypotheses were tested by residualized change regressions analogous to those reported above. Thereby, each model controlled for the participant’s score on the pre-experimental measure of the respective dependent variable.

Upon closer inspection, neither accuracy (β = 0.03, 95% CI [− 0.04, 0.09], *SE* = 0.03, *t*(124) = 0.74, *p* = 0.660) nor reaction time (β = 16.73, 95% CI [− 31.70, 65.17], *SE* = 24.46, *t*(119) = 0.68, *p* = 0.660), both obtained from the AX-CPT, displayed significant condition effects. Similarly, we could not observe significant condition effects in self-reported self-control (β = 1.33, 95% CI [− 0.48, 3.14], *SE* = 0.91, *t*(124) = 1.46, *p* = 0.592) and self-efficacy (β = − 0.13, 95% CI [− 2.40, 2.13], *SE* = 1.14, *t*(124) = − 0.12, *p* = 0.908).

## Discussion

In this paper, we introduced a new approach to supporting and training self-regulation. Concretely, we developed a desktop application that lets people set a goal for work/study sessions on their computer and then gives them feedback when they get distracted or return their focus back to the chosen task.

The results of our field experiment demonstrate that participants who received this feedback were more focused on their tasks during their working sessions, and they reported greater task motivation and self-control compared to participants in the control condition who did not receive the feedback. Specifically, after controlling for false discovery rate, we found that the experimental condition surpassed the control condition on six of eight task-specific performance indicators, including, crucially, actual behavioral focus. Further, the attentional feedback not only did not undermine subjective motivation, but actually increased task motivation and self-control.

Our study design may have prevented us from observing feedback-induced learning more systematically, as it did not include a baseline measurement period, and group differences in performance were already evident after the first training session. However, our exploratory follow-up analyses hint on evidence for reduced distraction frequency and length over time when receiving feedback, suggesting that our application holds potential to significantly enhance people’s ability to stay focused on their chosen tasks. By contrast, we did not find any significant effect on overall executive functions.

### Relationship to prior work

Previous work found that feedback is especially effective if it promotes self-assessment^25^. Compared to existing research^15^, our findings are based on a much larger sample and evaluate not only productivity but gain more in-depth insights into individual self-regulation by experience sampling dialogs present in the software. Beyond performance-related gains, we could show that our application’s feedback promotes self-assessment by reminding users of their self-set goals, continuously supporting self-evaluation, and monitoring of their progress towards these goals. Experiencing progress towards their individual goals might encourage users to increasingly strengthen their self-regulation competences as a valuable investment in long-term benefits and success. The substantial increase in task-related focus performance we observed also aligns with the overall positive effect of digital self-control tools reported by recent meta-analytic research^8^, even though the authors only found a small to medium effect on reducing distracted times over the seven included studies.

Our findings suggest that the software we developed enhances people’s capacity to pursue their own goals. It can therefore be seen as a cognitive prosthesis for self-regulation and goal-pursuit. While the cognitive prosthesis developed in previous work^2^ helped people choose a task and overcome motivational obstacles to getting started on it, the software presented in this article supports and trains the self-regulatory processes that are necessary to stay on task. Moreover, our approach can be seen as a novel form of boosting^52^. Previous work on boosting sought to improve people’s ability to make good decisions. Our software complements this work by boosting people’s capacity to follow through on their decisions.

### Limitations

A core problem of our field experiment were the quite high attrition rates between recruitment, the start of the experiment, and data submission (see Fig. 4). Given that the majority of dropout occurred between the pre-survey and the start of the intervention, we see a valid probability that these participants might not have been interested in the study at all or might have lacked the willingness to commit to a multiday study. While many studies do not report this proportion of participants at all, we decided to report them for reasons of transparency and to give other researchers working on similar questions a valid estimate of how many participants they can potentially expect to participate in a multiday study. Consequently, our actual dropout rate reduces to 43 participants out of 170 participants (25% out of everyone processing to the state of randomized group allocation), which is even lower compared to dropout rates reported for other internet-based longitudinal field experiments^40^.

To address the issue of dropout even better in future work, we will employ better recruitment and retention strategies and develop means to ensure robust data recording and reporting across all measures. A potential way might relate to introducing a bonus compensation for participation contingent on completing the study and providing reports on all measures. Tying in even earlier in the process, another approach could involve measures for building user’s trust in the system, for instance, by addressing factors investigated in the Unified Theory of Acceptance and Use of Technology (UTAUT^53^), such as perceived usefulness, ease of use, or behavioral control.

We may have been precluded from finding effects of differential growth that would reflect learning because we did not have measures of the outcomes before the first training session with our attention training software, and differences between conditions were already evident in the first session. However, the strong differences that were already evident at this point suggest that our application is effective at improving self-regulation. In addition, our evaluation only covered a short period of 12 days, hence, we cannot draw long-term conclusions—a general lack of evidence in the current study landscape on digital self-control tools^8^.

### Ethical challenges

When dealing with data-intense digital frameworks in educational settings, we need to be particularly aware of the related challenges^54^. In terms of the generated data, our application monitors people’s activities while they are working on their computer, meaning whether the program that is currently in focus is congruent with the activity that was selected to be done in the beginning of a focus session. Interaction signals related to mouse or keyboard activity are used to confirm the focused or distracted state. To avoid data misuse, the software applies masking procedures that only reveal the focused vs. distracted state without further details of use other than the target programs that were initially specified. Furthermore, the recorded data is transmitted via an encrypted archive, which is deliberately created by users to put them in control and support their agency and oversight. In principle, low focus scores could hint on prevailing attention disorders and bear negative consequences in school or workplace contexts when they get into the wrong hands. But the anonymized data storage and encrypted data transmission ensures protection against such risks.

Compared to currently existing programs for training cognitive skills, our software is more likely to transfer to the real world. As it gives people the opportunity to train with self-selected goals, it is highly connected to their daily lives. Hence, scaling up a software like this across various work and study contexts comes with many unknown effects. Following advice on evaluating experimental technology^55^, it is usually not possible to anticipate every conceivable consequence when introducing a novel technology to society. Hence, the proposed approach suggests treating this endeavor as a social experiment that adheres to the moral principles of non-maleficence, beneficence, respect for autonomy, and justice. The emerging framework entails monitoring benefits and risks very carefully and scaling back if unwanted consequences get obvious. These moral principles are at the core of designing and evaluating digital self-control tools that support users’ digital wellbeing in an ethically sound manner^8^. Hence, with the already implemented measures outlined above, we aimed at taking steps to avoid maleficent consequences by prioritizing data privacy, to ensure that users benefit in progressing towards their goals, to maintain users’ agency with regards to data sharing decisions, and to protect vulnerable user groups at schools and workplaces from unwanted impacts on their lives.

### Future directions

Our application opens up a broad avenue for future extensions related to the underlying feedback mechanism, sources of distraction, resumption support as well as applications in everyday life work and study contexts.

Following the idea of human-centered design, people should particularly benefit from personalized feedback. Besides changing the visual appearance of the software, for instance, in the direction of anthropomorphized pedagogical agents^56^, the feedback itself could be provided in an adaptive way. Applying evidence on the expertise-reversal effect^57^, machine learning algorithms could be embedded in the software framework to provide fading instructional guidance and allow users to effectively internalize the external feedback provided by the application. Thereby, future work could take additional parameters into account to derive optimal feedback values, for instance, mental fatigue, learning gains and subsequent task automation, or distractibility due to task saliency^33,58^. By using metrics such as the achieved focus performance or average training time, increasingly delayed and less frequent feedback would avoid people overly relying on constant assistance from the software (see^26^). Instead, they are encouraged to hone their own self-regulation skills and, consequently, can independently apply these skills across a wide range of situations.

To ensure a more comprehensive and rigorous evaluation of the usability aspects of such adaptive qualities in our software, future studies involving the application may adopt a more in-depth methodology. A proposed method advocates the integration of user and expert testing, guided by a set of evidence-based heuristics established in the same study^59^. The efficacy of this approach lies in its ability to leverage both user and expert perspectives, as evidenced by a demonstrated low overlap between issues identified by these two groups. Heuristics provide systematic guidelines for evaluation, contributing significantly to the robustness of the analysis. Additionally, the think-aloud approach in usability studies could be enhanced by incorporating AI assistants^60^. These AI assistant could analyze extensive information from audio and video streams, effectively clustering usability problems and user comments. This functionality would not only enhance the efficiency of the evaluation but also contribute to increased result reliability by basing the evaluation on a model.

Additional and more fine-grained insights in individual distractibility and sources of distraction would allow us to explore potential resumption support mechanisms, which could be embedded in the software to improve people’s skills in quickly recovering from distractions. Such support mechanisms could, for instance, provide memory cues for retrieving the previous task history or facilitate the search for cues in the visual environment^4,61^.

Our application might be able to help people stay focused in different real-world contexts. For instance, in the context of education, it could help students achieve their academic goals by improving their self-regulation skills and building beneficial workflows^62,63^. Because self-regulated learning becomes increasingly important from primary to secondary and tertiary education, training the necessary self-regulation skills early could be considered a valuable investment^64^. Tying in with prior work^65^, we further see added value in applying our software to support behavioral focus in the context of remote meetings, a frequent situation in international, distributed, and flexible working environments. In such settings, our optimal feedback could emphasize the value of invested resources for information capturing and sharing, thereby fostering the development of strategies to stay engaged in the discussion.

## Conclusion

Keeping our attention focused is a challenge that we all face across a variety of situations in our everyday lives. With rapid developments in user-centered technology design, we could combine findings on the effectiveness of goal setting and feedback with methods from machine learning to develop assistive technologies that help people stay focused on their goals. In this article, we reported a multi-day field evaluation of one such technology. Overall, our results suggest that the current version of our application is an important step towards empowering people to improve their ability to focus on their goals and become more effective.

### Supplementary Information

Supplementary Information.

## Data Availability

An anonymized version of the dataset created in the reported study can be accessed via https://osf.io/8f6h/.
