# Supplementary material for: Optimal feedback improves behavioral focus during self-regulated computer-based work
Source: Sci Rep. 2024 Feb 7;14:3124. doi: 10.1038/s41598-024-53388-3 (PMC10850098; doi:10.1038/s41598-024-53388-3)
Supplement: Supplementary file 1 — Supplementary Information. [file 41598_2024_53388_MOESM1_ESM.docx]

Optimal feedback improves behavioral focus during self-regulated computer-based work

Maria Wirzberger^1, 2, 3,^ *, Anastasia Lado^1, 2, 3^ , Mike Prentice^2^, Ivan Oreshnikov^2^, Jean-Claude Passy^2^, Adrian Stock^1, 2^ & Falk Lieder^2^

^1^University of Stuttgart, Stuttgart, Germany

^2^Max Planck Institute for Intelligent Systems, Tübingen, Germany

^3^ These authors contributed equally.

*Corresponding author: Maria Wirzberger

Email: [maria.wirzberger@iris.uni-stuttgart.de](mailto:maria.wirzberger@iris.uni-stuttgart.de)

Phone: +49 711 685 811 76

University of Stuttgart

Interchange Forum for Reflecting on Intelligent Systems

c/o Teaching and Learning with Intelligent Systems

Geschwister-Scholl-Str. 24D

70174 Stuttgart

Germany

# Supplementary material

## A1. Optimal metacognitive feedback

Exerting cognitive control is often beneficial and always costly. In some situations, the benefits of cognitive control outweigh the costs, but in other situations the costs of cognitive control outweigh its benefits. People therefore have to decide when it is worthwhile to exert cognitive control and when it is not. When a person pursues a goal over an extended period of time, they face this decision not just once, but almost continuously. Therefore, the value of exerting cognitive control at one time depends on whether they will later continue to exert control or let loose and get distracted. Following prior work [1], we model this sequence of decisions as a finite-horizon Markov Decision Process (MDP; [2]). A finite-horizon MDP is defined by the set of possible states that an agent can be in, the set of actions the agent can take, the conditional probabilities of transitioning from one state to another depending on the action taken ($T$), a reward function ($r$), and for how long a task is performed ($h$). Hence, the challenge of attention control can be modeled by the MDP

$$M=\left( \left\{ s_{f},s_{d} \right\}, \left[ 0,1 \right],T,r,h \right). (1)$$

The states $s_{f}$ (focused) and $s_{d}$ (distracted) correspond to focusing on the chosen goal versus getting distracted by irrelevant stimuli. The available actions $c\in[0,1]$ are control signal intensities (ranging from 0% to 100%) with which top-down attention can be directed towards the task goal. In each time step, we can observe one of four potential state transitions: being focused and staying focused, being focused and getting distracted, being distracted and getting focused again, and being distracted and staying distracted. The transition function $T(s_{t},s_{t+1};c_{t})$ specifies the probabilities of these four state transitions depending on the chosen control signal intensity $c_{t}$. Based on the resulting state transition, the reward function

$$r\left( s_{t},c_{t},s_{t+1} \right)= I_{s_{t}= s_{t+1}}\cdot r_{s_{t}}-cost\left( c_{t} \right), (2)$$

encodes the value generated minus the cost of control (cost$(c_{t})$). The identity function $I_{s_{t}= s_{t+1}}$ is used to express the assumption that the value of pursuing the (un)intended activity (i.e., $r_{s_{t}}$) is not obtained during task-switching ($s_{t}\neq s_{t+1}$). Following previous work (e.g., [1][3]) the cost of control can be described by an exponentially increasing function of the control signal intensity, that is

$$cost\left( c \right)=exp\left( \frac{c}{2} \right) -1. (3)$$

Subtracting 1 ensures that not exerting any cognitive control has a cost of 0. The horizon $h$ is the length of the task in time steps.

A powerful way to foster people’s skills to stay focused and inhibit distractions is to give them feedback that conveys the value of staying focused. Machine learning researchers have discovered that certain types of feedback can accelerate the rate at which reinforcement learning agents acquire complex motor skills [4]. This line of work highlights that to be beneficial, the feedback the agent is given has to obey the principle of reward shaping. Existing research [5] applied this principle to cognitive training and found that the optimal feedback signal is

$$FB\left( s,c \right)=Q_{meta}\left( s,c \right)-{max_{c} Q}_{meta}\left( s,c \right) , (4)$$

where $Q_{meta}\left( s,c \right)$ is the value of performing the cognitive operation $c$ in the internal state $s$. This metacognitive feedback was highly effective at teaching people optimal planning strategies [5][6]. The rationale of this feedback signal is to provide immediate rewards that accurately communicate the long-term value of individual cognitive operations. In the context of attention control, the cognitive operations are cognitive control signals and $Q\left( s,c \right)$ is the Expected Value of Control (EVC; [3]). Plugging this definition into *Equation 4*, we find that the optimal metacognitive feedback signal for attention control training is

$$FB\left( s_{t},c_{t} \right)=EVC\left( s_{t},c_{t} \right)- max_{c}EVC\left( s_{t},c \right) . (5)$$

Considering the current state $s_{t}$and control signal strength $c_{t}$, our feedback communicates the value of the invested attention control compared to the value of the best attention control signal that the person could have chosen. As the control signal strength $c$is not directly observable, we have to infer it from people’s behavior. This can be done by performing Bayesian inference.

We compared different methods for estimating the control signal intensity, either by using the maximum a posteriori probability (MAP) estimate of $c_{t}$ or by computing the expected value of $c_{t}$ under its posterior distribution given the observed behavior. The prior distribution was either a uniform distribution, with equal probability for all control signal strengths, or a Beta(2,2) distribution, which assigns higher probability to intermediate control signal intensities. To determine which of the four ways of estimating the control signal intensity leads to the most beneficial feedback signals, we conducted computer simulations. In these simulations, we used the Learned Value of Control (LVOC) model [1] to predict how well people would learn from the feedback resulting from either way of estimating the control signal intensity. Our simulation results revealed that feedback derived from MAP estimation with a Beta(2,2) prior leads to significantly faster learning. We therefore used this approach to estimate the control signal intensity. Plugging the resulting estimates into *Equation 5* and shifting all feedback signals so that staying focused yields a reward of 0.1 produced the rewards $R_{sim}$ shown in *Supplementary table S1.*

To translate the reward per state-transition ($R_{sim}$) into the number of points that our application should award or deduct per minute of staying focused/distracted ($R_{imp}$), we measured how long each state transition typically took. We then divided the number of points per time step by the measured average duration of a state transition in minutes, considering that reminders for staying distracted were shown every 7 s. To determine the number of points that our application should award for getting distracted and regaining focus, we multiplied the simulated point values by 2. The resulting point values that were implemented into our app are shown in the last column of *Supplementary table S1* ($R_{imp}$). Those values show that the app’s feedback was most intense when the user got distracted, and second most intense when a distracted user reoriented their attention back to their chosen task. The number of points that the user lost for being distracted was slightly higher than the number of points that they gained for staying focused for the same amount of time.

**Supplementary table S1.**

*Feedback signal strengths for state transitions.*

| ***Event*** | $R_{sim}$ | $R_{imp}$ |
| --- | --- | --- |
| $s_{f},s_{f}$ | 0.3910 points | 9.6240 points/min |
| $s_{f},s_{d}$ | -1.2298 points | -2.4596 points/transition |
| $s_{d},s_{f}$ | 0.4873 points | 0.9746 points/transition |
| $s_{d},s_{d}$ | -0.4434 points | -10.9140 points/min^a^ |

*Note.* ^a^ While the user remained distracted, they lost 1.2733 points every 7 seconds.

Following the definition of the MDP, the focus score can be calculated from these state transitions and the related rewards as follows:

$focus score = n_{s_{f},s_{f}}\cdot r_{s_{f},s_{f}}+n_{s_{f},s_{d}}\cdot r_{s_{f},s_{d}}+ n_{s_{d},s_{f}}\cdot r_{s_{d},s_{f}}+ n_{s_{d},s_{d}}\cdot r_{s_{d},s_{d}}$, (6)

where $n_{a,b}$ is the number of times the user transitioned from state $a$ to state $b$ and $r_{a,b}$ is the corresponding value of $R_{imp}$ shown in *Supplementary table S1.*

## A2. Kalman filter

Generally, the Kalman filter is a group of mathematical methods for estimating underlying system states from observations while removing measurement errors. Building on the default settings of the pykalman module [7], our Kalman filter calculates state means and covariances from the normalized focus scores of all existing focus sessions. Initial state mean and covariance were derived from an empirical study [8].

## A3. Deviations from pre-registration

We refrained from using the normalized focus score, as we encountered conceptual inaccuracies in the technical implementation. While the initial rationale behind normalizing the obtained point score was to ensure comparability between sessions of different lengths, compared to the unnormalized focus score the current implementation of the normalized focus score gives less weight to events that occurred in longer sessions than to events that occurred in shorter sessions. Hence, we can consider the unnormalized focus score as a more reliable measure of people’s actual ability to stay focused.

In addition, our original plan involved also inspecting the time people spent productively and unproductively, measured by using a commercial time tracking software. Unfortunately, we observed a substantial lack of data points, emerging from various reasons (e.g., hesitation to use this software at all, using the software but not providing data, loss of data due to a software bug). Consequently, we could only gather data from a small proportion of our sample (*n* = 14 participants), which did not provide a sufficient base for statistical hypothesis testing. Hence, we did not pursue those hypotheses here.

## References

1. Lieder, F. et al. Rational metareasoning and the plasticity of cognitive control. *PLoS Comput. Biol.* **14**, e1006043 (2018).
2. Sutton, R. S. & Barto, A. G. *Reinforcement Learning: An Introduction* (The MIT Press, 2018).
3. Shenhav, A., Botvinick, M. M. & Cohen, J. D. The expected value of control: an integrative theory of anterior cingulate cortex function. *Neuron* **79**, 217–240 (2013).
4. Ng, A. Y., Harada, D. & Russell, S. Policy invariance under reward transformations: Theory and application to reward shaping. In *Proceedings of the 16^th^ Annual International Conference on Machine Learning* 278–287 (Morgan Kaufmann, 1999).
5. Callaway, F. et al. Leveraging artificial intelligence to improve people’s planning strategies. *Proc. Natl. Acad. Sci.* **119**, e2117432119 (2022).
6. Lieder, F. et al. A cognitive tutor for helping people overcome present bias. In *The 5^th^ Multidisciplinary Conference on Reinforcement Learning and Decision Making* (2019).
7. Duckworth, D. *pykalman 0.9.2 documentation* (2012). Available at: https://pykalman.github.io/ (Accessed: September 14, 2023).
8. Wirzberger, M. et al. How to navigate everyday distractions: Leveraging optimal feedback to train attention control. In *42^nd^ Annual Meeting of the Cognitive Science Society* 1736 (Cognitive Science Society, 2020).
